# Cascade screening for beta-thalassemia in Pakistan: development, feasibility and acceptability of a decision support intervention for relatives

**DOI:** 10.1038/s41431-021-00918-6

**Published:** 2021-06-14

**Authors:** Shenaz Ahmed, Hussain Jafri, Yasmin Rashid, Yasmin Ehsan, Shabnam Bashir, Mushtaq Ahmed

**Affiliations:** 1 Leeds Institute of Health Sciences, University of Leeds, Leeds, UK; 2 Thalassaemia Society Pakistan, Lahore, Pakistan; 3 Health Department Punjab, Lahore, Pakistan; 4 Punjab Thalassaemia Prevention Project, Lahore, Pakistan; 5 Yorkshire Regional Genetics Service, Leeds, UK

**Keywords:** Preventive medicine, Ethics

## Abstract

The government-funded ‘Punjab Thalassaemia Prevention Project’ (PTPP) in Pakistan includes cascade screening for biological relatives of children with beta-Thalassaemia Major (β-TM). However, there is low uptake of cascade screening. This paper presents the (i) development of a paper-based ‘decision support intervention for relatives’ (DeSIRe) to enable PTPP Field Officers to facilitate informed decision making about carrier testing, and (ii) assessment of the feasibility and acceptability of the DeSIRe. The intervention was developed using the International Patient Decision Aids Standards quality criteria and Ottawa Decision Support Framework. Twelve focus groups were conducted (September and October 2020) to explore the views of healthcare professionals (HCPs) and relatives of children with β-TM, in six cities. The focus groups were attended by 117 participants (60 HCPs and 57 relatives). Thematic analysis showed that the DeSIRe was considered acceptable for supporting relatives to make informed decisions about cascade screening, and potentially feasible for use in clinical practice. Suggestions for changing some words, the structure and adding information about how carrier testing relates to consanguineous marriages will enable further development of the DeSIRe. Participants generally welcomed the DeSIRe; however, they highlighted the perceived need to use more directive language, hence showed a cultural preference for directive genetic counselling. The findings highlight challenges for researchers using western theories, frameworks, policies and clinical guidelines to develop decision support interventions for implementation more globally. Future research is needed to evaluate the use of the DeSIRe in routine practice and whether it enables relatives to make informed decisions.

## Introduction

Beta-Thalassaemia Major (β-TM) is the most common genetic disorder in Pakistan, with an estimated nine million carriers, 40,000 children registered as transfusion-dependent and 5000–9000 children born annually with the condition [1]. The life expectancy of individuals with β-TM has increased in many countries to the fifth decade, but remains about 10–12 years in Pakistan due to limited resources and limited availability of safe blood for transfusions, where the risks of acquiring HIV and Hepatitis B and C are high [2]. Undiagnosed and untreated children usually die in early childhood [2], although there is a paucity of epidemiological research on infant mortality due to β-TM in Pakistan, so the true burden of β-TM is most likely an underestimation [3]. Overall, Pakistan has a huge economic and welfare burden due to β-TM.

Effective implementation of cascade screening to enable informed marital and reproductive decisions can help to reduce the burden of β-TM in Pakistan.

Suggestions for interventions to facilitate cascade screening include: letters from healthcare professionals (HCPs) for relatives; professional encouragement of consultants to share information; and supporting communication using written information aids [4]. However, these western approaches are unlikely to succeed in Pakistan, because parents of children with β-TM tend to have poor knowledge of the inherited nature of their child’s condition, and low literacy levels [5]. Thus, these parents are unlikely and often unable to communicate information about cascade screening. Also, research in other countries shows that even when people understand the implications for family members, they find it difficult to discuss genetic risk due to family dynamics [4].

Cascade screening for β-TM is recognised as a priority in the government-funded provincial ‘Punjab Thalassaemia Prevention Project’ (PTPP). Therefore, parents of children with β-TM attending thalassaemia clinics are approached by PTPP Field Officers (FOs) to discuss carrier testing of relatives. Parents willing to support cascade screening then inform their relatives and arrange individual or group meetings at their home for them to meet the FO, who then provides information about β-TM and genetic inheritance, and offers cascade screening. While FOs are responsible for facilitating cascade screening for β-TM, they do not have information resources specifically for relatives. This may explain the low uptake of cascade screening by relatives. Furthermore, we are not aware of the availability of any resources for cascade screening for β-TM (or any other genetic condition) in Pakistan or in any other low- or middle-income country. Therefore, there is a need for an intervention (containing accessible information) to enable HCPs to facilitate informed decision making about carrier testing in relatives of affected families.

The aims of this study were to (i) develop a ‘decision support intervention for relatives’ (DeSIRe) prototype for use by PTPP FOs to enable them to facilitate informed decision making about cascade screening, and (ii) explore the views of relatives and HCPs to assess the acceptability and feasibility of the DeSIRe.

## Materials and method

This study was approved by the Ethics Review Committee at Fatima Jinnah Medical University, Lahore, Pakistan and by the School of Medicine Research Ethics Committee, University of Leeds, UK.

### Development of DeSIRe prototype

The DeSIRe was developed as a paper-based tool for use by PTPP FOs in face-to-face, individual or group consultations. The aims of the DeSIRe were to: (1) provide information to relatives about the condition, recessive inheritance and implications of being a thalassaemia carrier; (2) support FOs’ consultations with relatives; and (3) assist relatives in making decisions about carrier testing that are consistent with their values. The DeSIRe prototype was initially developed in English by a team consisting of a social scientist (SA) and two senior genetic counsellors (HJ and MA), each with over 20 years of research and clinical expertise on the topic—all bilingual in English and Urdu. This team then translated the DeSIRe to Urdu, for feedback and use in Pakistan. We are not aware of any readability tests for documents in Urdu, so the DeSIRe was developed using strategies known to make text more readable. All subsequent revisions to the DeSIRe were in Urdu only.

The DeSIRe was developed using the International Patient Decision Aid Standards guideline, which provides a framework for the development of a decision support intervention [6]. Accordingly, the DeSIRe prototype included balanced information, probabilities of decision outcomes, clarification of individual values and guidance to enable deliberation of the carrier testing decision. The development process was systematic, ensuring the DeSIRe was based on a synthesis of relevant research evidence and transparency. Furthermore, an iterative review-revise process by the research team and study’s Expert Panel based in Pakistan was used until consensus was reached about the content of the DeSIRe. The Expert Panel of key stakeholders included two parents and two relatives of children with β-TM, three PTPP FOs, a member of the Thalassaemia Society Pakistan NGO, a genetic counsellor, a haematologist and an obstetrician (the latter two clinicians were also decision makers in the PTPP).

The Ottawa Decision Support Framework (ODSF) was used as the conceptual framework to guide the content and structure of the DeSIRe prototype. The ODSF is informed by several psychological and decision-making theories, including expected utility theory, decisional conflict theory and social support theory [7]. The ODSF suggests that identification of individuals’ decisional needs can enable provision of personalised decision support, enabling informed and value-based health decisions, hence improved decisional quality [8]. The ‘decision support’ components of the ODSF were used to guide the content and structure of the prototype to enable relatives to: (a) recognise the need to make a decision; (b) understand evidence-based information, people’s preferences and probabilities; (c) consider factors affecting the decision; (d) clarify values and identify preferences based on these values; and (e) form a plan of action. Accordingly, the DeSIRe included evidence-based information about: β-TM, how it is inherited, including the implications of being a thalassaemia carrier, explicit illustration of recessive inheritance, and the probability of carriers having a child with β-TM; risks and benefits of carrier testing for relatives; a values clarification exercise; encouragement for relatives to make their own decision according to their own personal values; and information on how to go about having carrier testing.

The DeSIRe prototype, a leaflet formatted by a graphic designer, printed in colour (see Supplementary information), was used to obtain feedback on acceptability and feasibility.

### Accessibility and feasibility of the DeSIRe

#### Participants and recruitment

The two participant groups included relatives of children with β-TM and HCPs associated with the work of the PTPP. The inclusion criteria for relatives were: aged 18+ years, able to read, have at least one biological relative in the family with β-TM and able to consent to participate. Convenience sampling was used to recruit participants via six PTPP Regional Centres that covered 36 districts of the Punjab province–Lahore, Rawalpindi, Bahawalpur, DG Khan, Multan and Faisalabad. Respective district FOs initially contacted parents of a child with β-TM (registered with the PTPP), asking them to verbally pass on information about the study to their biological relatives. Each of these parents provided telephone numbers of up to two relatives consenting to receive further information. FOs approached and consented these potential participants via telephone. Interested participants were provided with details of the focus group date, time and venue. Moreover, FOs also initially contacted HCPs, and those interested were consented to the study by the researchers.

Twelve focus groups were conducted in the six PTPP Regional Centres, one with each participant group, during September and October 2020. Heterogeneity within the groups was based on age, gender, and education (relatives) or profession (HCPs).

#### Data collection

Based on the existing literature on evaluation of decision support interventions, a semi-structured facilitator’s guide was developed to include open-ended questions on: readability, appearance and length, perceptions of specific content, perceptions on the use of the DeSIRe to support decision making, perceptions of feasibility and overall satisfaction. The focus groups were held in hotel meeting rooms, where participants were able to observe social distancing (due to the COVID-19 pandemic). Three qualitative researchers (one facilitator and two moderators) conducted each focus group, following provision of the DeSIRe to each participant, and a description of its intended use in the PTPP. The focus groups lasted approximately 90 min, with nine or ten participants. All focus groups were conducted in Urdu, audio recorded, translated and transcribed in English by two bilingual researchers.

#### Data analysis

Transcripts were managed in Nvivo 12 (software) and analysed using a combination of inductive and deductive thematic analysis [9]. SA developed a coding framework based on the research aims, facilitators guide and initial analysis of two transcripts using principles of grounded theory (open, axial and selective coding). This framework was used to code all the transcripts. Codes emerging from the data during analysis of subsequent transcripts were added to the coding framework. Data analysis also involved consistent cross-referencing between the participants within and between focus groups for similarities and differences. All data were analysed by the same experienced qualitative researcher (SA), who discussed the coding framework and themes with HJ and MA at length, then refined and discussed again to ensure consistency in interpretation of the data. During analysis, differences by participant group, gender and educational attainment were explored but not found.

## Results

The 12 focus groups included a total of 117 participants, 57 relatives and 60 HCPs. Demographic characteristics of the relatives and HCPs are presented in Table 1. The focus groups with HCPs included 26 PTPP FOs, 5 genetic counsellors, 10 gynaecologists, 8 nurses, 5 medical officers and 6 other affiliated HCPs.

**Table 1:** Demographic characteristics of the focus group participants.

		Relatives *N* = 57	Healthcare professionals *N* = 60
Gender	Male Female	30 (53%) 27 (47%)	32 (53%) 28 (47%)
Age (years)	Mean (SD)	32.3 (8.5)	30.4 (9.1)
Participants’ education	Up to matriculation level^a^ Above matriculation level	32 (56%) 24 (44%)	– 60 (100%)

^a^Matriculation level is equivalent to the UK GCSE level at around age 16 years.

### Acceptability and enhancing clarity

Participants generally evaluated the paper copy of the DeSIRe positively, including its appearance, length and format, although some participants suggested increasing the font size. The amount and type of information in the DeSIRe was considered appropriate, though participants made suggestions for some additional information and changes to the structure. For example, they believed it was important to highlight that the carrier test is free of charge at the outset, to ensure that relatives do not disengage with the intervention due to concerns about the cost of testing:

“*…add that this test will be free… If you write it at the beginning, people will take interest and read on. Otherwise, they’ll put it to one side” (Female relative, Bahawalpur)*

The first subsection containing the description of β-TM was considered sufficient and informative, although participants suggested adding a sentence on bone-marrow transplant as a potential cure for β-TM for completion of information. They also suggested adding a separate subsection on ‘What is a thalassaemia carrier?’, before presenting information on inheritance. This editing was believed to (i) enable a better understanding of thalassaemia carriers and how they differ from individuals with β-TM, and (ii) prepare relatives for the subsequent information on genetic inheritance.

Participants also suggested that in addition to the information and image on the inheritance pattern for two thalassaemia carriers, the inheritance pattern for a thalassaemia carrier and non-carrier should also be included. This was believed to enable HCPs to better explain (and relatives to better understand) recessive inheritance and the relevance of carrier testing:

“*…this picture would help relatives understand why thalassaemia may be in their family, even when they don’t have a child with the condition.” (Female HCP, Multan)*

Participants did not raise any concerns about the brief content on consanguineous marriages. Instead they strongly recommended the addition of a separate subsection to highlight both the chances of having a child with β-TM when marrying (i) within the family, because of the high rate of consanguineous marriages in Pakistan, and (ii) outside of the family, because of the high carrier frequency in the Pakistani population:

“*…people think they don’t need testing if they marry out of the family… make it clearer they can have a child with thalassemia in both cases… they need testing.” (Male relative, Multan)*

Participants generally agreed that the DeSIRe was easy to read and understand. Suggestions for improving readability focused mainly on words used in the ‘inheritance’ subsection: replace *naquis* (defective) with *kharaab* (faulty) and *maroosi* (hereditary) with *khandaani* (familial). The English word gene(s) was highlighted, particularly by relatives, as difficult to understand. But after in-depth discussion of alternative words, there was agreement that it was important to use the English word followed by a lay description:

“*People don’t know what this (gene) is, but you have to call it what it is because otherwise you would be wrong.” (Male HCP, Faisalabad)*

In addition, the presentation of probability information in the form of both percentages and ratios was considered confusing. The use of percentages only was recommended:

“*…1 in 4 …people think that one of their children has this disease so the next 3 won’t. This is a huge misconception …change it to 25% chance.” (Female HCP, Lahore)*

Furthermore, a number of HCPs believed the inheritance pattern image would be difficult to understand for people with low literacy levels, but its inclusion was considered important for HCPs to use to explain recessive inheritance in a more interactive way, as would be expected in genetic counselling.

The addition of a picture of a child with β-TM to improve readability was also discussed. Some participants believed it was important to show the severity of the condition through images of a child having treatment (blood transfusion or chelation therapy). However, others believed such pictures would raise ethical challenges, given that children with β-TM do not have any obvious dysmorphic features:

“*…scaring people is necessary if we want to get rid of this condition… so people should have a bit of a shock when they see the picture” (Male relative, Rawalpindi)*

“*No, it’s not ethically okay. Sometimes… we have to ask, ‘are you really thalassemic?’ …you have to give them balanced information.” (Male HCP, Multan)*

### Perceptions of balanced information and non-directiveness

Participants’ in all the FGs recognised that the DeSIRe contained balanced and non-directive information, which enabled relatives to decide whether or not to have carrier testing. They agreed with the provision of detailed information to support decision making about thalassaemia carrier testing. However, their suggestions for further changes show their preference for directive language to emphasise the importance of carrier testing:

“*It’s very important to tell all relatives that it’s very important to have this test before getting married.” (Male relative, DG Khan)*

“*Don’t give them options… make it clear the tests are important…” (Male HCP, DG Khan)*

Some participants believed carrier testing was in the best interest of relatives and explained that a balanced, non-directive approach could lead them to decline testing without understanding its significance. Therefore, they suggested a strong steer was needed to influence participants to opt for carrier testing:

*You should use emotional blackmail by adding that ‘you’re responsible if your child has thalassaemia major’.” (Male relative, Lahore)*

“*We have to scare them… tell them the problems they’ll face.” (Female HCP, Multan)*

Also, HCPs clarified that relatives expect a directive approach because it is the cultural norm and giving them a choice could lead to confusion about the importance of testing:

“*People ask us what they should do. Our people won’t get it.” (Female HCP, Bahawalpur)*.

### Perceptions of clarifying values

Participants, particularly HCPs, believed the values clarification exercise was unnecessary. This exercise was included to help relatives clarify their personal values and how they relate to cascade screening, but participants believed it would be confusing for relatives, especially those with poor/no literacy and accustomed to directive approaches:

“*we don’t need to give them options… don’t get them involved in all this detail. … they are not literate and can’t consider the information in this way. They will get confuse” (Male HCP, Bahawalpur)*

“*If we ask them “Are you sure that the decision you are making is the best decision for you?” they will get confused… worried.” (Female HCP, Lahore)*

Some participants were dismissive of this exercise because they believed supporting relatives to consider their values against carrier testing would lead to them doubting the importance of carrier testing and deciding against opting for it:

“*Don’t raise doubts in his mind by adding these questions. Add a line like ‘thalassemia carrier testing today, not tomorrow’.” (Female relative, Lahore)*

“*People don’t know the implications of testing and by telling them that they may feel this or that, they will have concerns about getting tested.” (Female HCP, DG Khan)*

In particular, FOs believed their role was to encourage relatives to opt for testing because their performance was partly based on the uptake rate of carrier testing. They clarified that the values clarification exercise could result in relatives declining testing, hence would reflect negatively on their performance. Therefore, they believed the exercise should be deleted from the DeSIRe:

“*Our objective is that they should get tested. …it doesn’t make sense to pose these questions…” (Male HCP, Lahore)*

Nevertheless, other participants believed that the provision of information in itself indicated the importance of testing and that it was important to give relatives the opportunity to consider their values:

“*People will think there’s a reason for all the information given… they will understand testing is important. So it’s okay to ask these questions.” (Female relative, Rawalpindi)*

### Perceived need and enhancing feasibility of the DeSIRe

Overall, most participants believed the DeSIRe was a valuable resource that met relatives’ information needs. It was believed to enable a better understanding of β-TM, recessive inheritance and the relevance of carrier testing for relatives. Relatives added that they could use the DeSIRe to communication with other relatives to further raise awareness about the condition:

“*…families with experience of thalassemia will definitely read this… uncles, aunts and first-degree relatives…” (Male HCP, Multan)*

“*I didn’t know all of this… now I can use it (DeSIRe) to talk to other relatives. Then they will talk to others and this will help even more people to learn about thalassaemia.” (Female relative, Rawalpindi)*

Participants suggested that the DeSIRe should be used by FOs to complement counselling for groups of relatives. There was a consensus that the DeSIRe should be read aloud by FOs, preceded by encouragement for relatives to ask questions and request further explanation where necessary. Participants further clarified that FOs should identify individuals who can read within groups of relatives, provide them with a copy of the DeSIRe, and ask them to support communication when needed:

“*…there’s usually at least one person in a family who can read and understand this much… give at least one copy of this to each family… but it should be explained by you first.” (Male relative, Rawalpindi)*

“*It should be read out to everyone by the FO or the educated person in the family.” (Female relative, Multan)*

Furthermore, participants agreed that relatives should keep copies of the DeSIRe to enable them to go through the information in their own time.

## Discussion

The DeSIRe was considered acceptable for supporting relatives to make informed decisions about cascade screening, and potentially feasible for use in clinical practice. Suggestions for changing some words, the structure and adding information will enable further development of the DeSIRe. Participants generally welcomed the information, but expressed preferences for the use of more directive language to support informed decision making. Such preferences highlight challenges for researchers using western theories, frameworks, policies and clinical guidelines to develop decision support interventions for implementation more globally.

Regarding the content of the DeSIRe, participants believed information about the free availability of carrier testing at the outset was important. This may appear to bias decision making towards testing, but people in Pakistan often pay for healthcare and may be concerned about costs. Reduced user costs are associated with better health outcomes in low- and middle-income countries [10]. Therefore, informing relatives at the outset that carrier testing is free is key to enhancing their receptiveness to the DeSIRe.

Moreover, the DeSIRe prototype did not explicitly contain information about adverse health risks associated with consanguineous marriages, because this can be considered culturally insensitive and stigmatising, particularly for Pakistani populations living in western countries [11–13]. However, participants in our study highlighted the importance of a separate subsection within the DeSIRe to explain the significance of carrier testing when marrying within the family. Participants also suggested expanding this to include the implications of marrying out of the family, because of the high carrier frequency within the population and to address misconception that β-TM is restricted to people marrying within families. Similar to others [14], our findings show that information on the implications of marrying both within and out of the family is culturally sensitive and acceptable in countries where the practice of consanguineous marriages is a social norm.

During discussions about comprehension, participants largely agreed that the DeSIRe was easy to read and understand for a population with low literacy rates. However, key concepts in genetic counselling, such as ‘inheritance’ and ‘genes’, were still considered difficult to understand. Nevertheless, the use of these words in the DeSIRe is important to enable an understanding of the key messages about genetic inheritance, and to ensure completeness of information in the DeSIRe. Literal translation of such concepts is unlikely to be useful because of their unfamiliarity to potential readers and lack of use in everyday language [15]. Instead, use of English words was considered acceptable if accompanied with a brief explanation using everyday language and verbal explanation by the FO [15]. These findings show that decision support interventions for populations with low literacy rates should be developed to support existing communication by HCPs.

Moreover, the DeSIRe included numerical risk information using multiple formats to maximise understanding [16]. However, participants found the inclusion of both percentages and proportions confusing, and expressed a preference for percentages only. Further research is needed on which formats of numerical risk information are most appropriate for individuals with low numeric literacy in low- and middle-income countries.

More generally, participants agreed that the DeSIRe contained balanced information that allowed relatives to make decisions themselves. However, similar to other studies [17, 18], participants expressed a preference for the use of more directive language and images to influence relatives to opt for carrier testing. Such use of directive language would go against ‘non-directiveness’, a central ethical principle for genetic counselling [19–21]. This principle emphasises respect for patients’ autonomy and the importance of not leading individuals ‘to make particular decisions or choices… but to help them to make the best decisions for themselves and their families as judged from their own perspectives’ [19]. Such differences in cultural perceptions of patient autonomy and use of directive approaches for decision making raise ethical challenges to developing balanced interventions for implementation more globally. Despite preferences for directiveness, the DeSIRe will remain balanced to ensure alignment with key ethical principle of genetic counselling to support autonomous decision making. Also, training for HCPs on how to use the DeSIRe in practice will focus on ‘active non-directiveness’ [22], to enable them to not only provide information, but also involve relatives in decision making by raising and discussing various factors using the ‘values clarification exercise’.

Similarly, participants were apprehensive about the inclusion of the ‘values clarification exercise’ in the DeSIRe, because this was also believed to enable relatives to opt out of carrier testing. However, supporting autonomous decision making involves enabling individuals to make decisions in accordance with their own values [23]. Accordingly, evidence suggests decision support interventions should include an explicit values clarification exercise, as it enables individuals to recognise their own values and to share these with HCPs [24]. Moreover, such an exercise can result in decisions that better align with individuals’ values [25, 26], and reduce decisional regret mainly in people with lower health literacy [27]. Thus, while participants’ concerns in this study are recognised, the values clarification exercise will remain part of the DeSIRe. Accordingly, training for HCPs to use the DeSIRe will include justification for this exercise.

Moreover, HCPs preferences for directive approaches are understandable given the context in which they work, where uptake of carrier testing is a key performance indicator. Therefore, in line with international consensus for population genetic screening programmes [28], at policy and practice levels, emphasis should be placed on ‘informed choice’ as the aim of genetic screening programmes, not increased uptake. Accordingly, training for HCPs providing genetic counselling should focus on ‘active non-directiveness’ to safeguard individual autonomy and choices [22, 28].

### Strengths and limitations

A strength of this study is that, to the best of the authors’ knowledge, this is the only investigation with relatives of children with β-TM in a low- middle-income country focusing on their needs in relation to the making of decisions about cascade screening. Also, unlike related research in this field, the qualitative approach enabled participants to discuss complex views in detail. Moreover, to ensure transferability of the results, the study included relatives with diverse demographics and a range of HCPs, from six different cities in the Punjab region of Pakistan.

This study has limitations. To explore views about accessibility of the DeSIRe, an inclusion criterion for relatives was ability to read. While individuals with low levels of literacy were included (defined as having up to ‘Matriculation level’ education, equivalent to the UK GCSE level), further research is needed with relatives unable to read. Also, relatives were asked to read the DeSIRe during the focus groups, yet the intervention was developed for use by HCPs. Further research is needed to pilot-test the DeSIRe in real-life settings to support actual decision making about cascade screening.

### Conclusions

The DeSIRe meets the information needs of relatives for cascade screening for β-TM in Pakistan. Its use in the PTPP is feasible and acceptable to support relatives’ decision making about carrier testing. Participants’ feedback will be used to improve the DeSIRe by further simplifying and clarifying its content, and to develop a training workshop for HCPs to implement the intervention. Further research to pilot-test the revised DeSIRe will enable evaluation of barriers and facilitators to its use in routine practice. Subsequent studies will evaluate whether relatives’ decision making is informed and value based following the use of the DeSIRe.

## Supplementary information

The Decision Support Intervention for Relatives: Prototype
