# Supplementary figures and images for: Cascade screening for beta-thalassemia in Pakistan: development, feasibility and acceptability of a decision support intervention for relatives
Source: Eur J Hum Genet. 2021 Jun 14;30(1):73–80. doi: 10.1038/s41431-021-00918-6 (PMC8200315; doi:10.1038/s41431-021-00918-6)

DeSIRe prototype, page 1


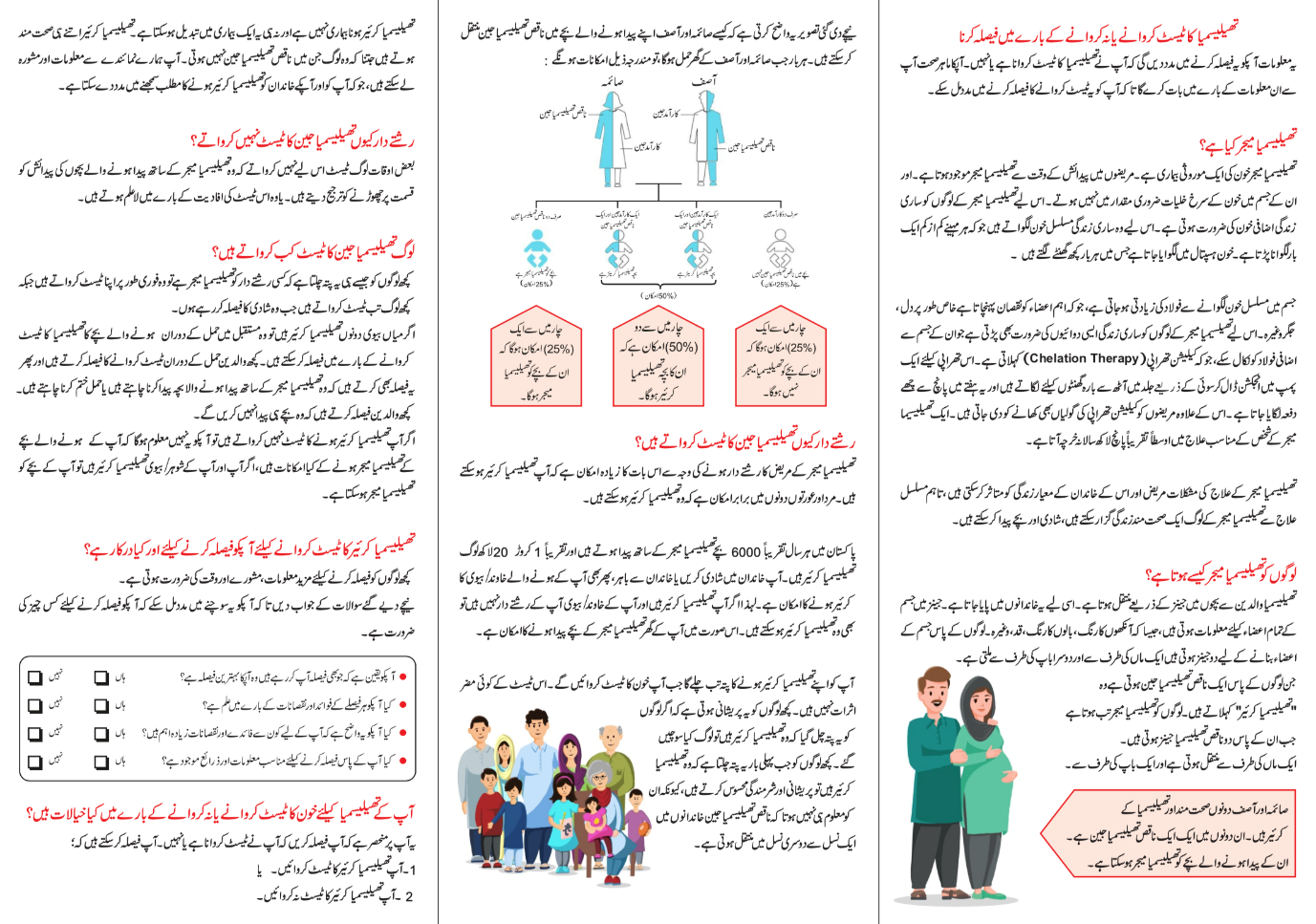


DeSIRe prototype, page 2


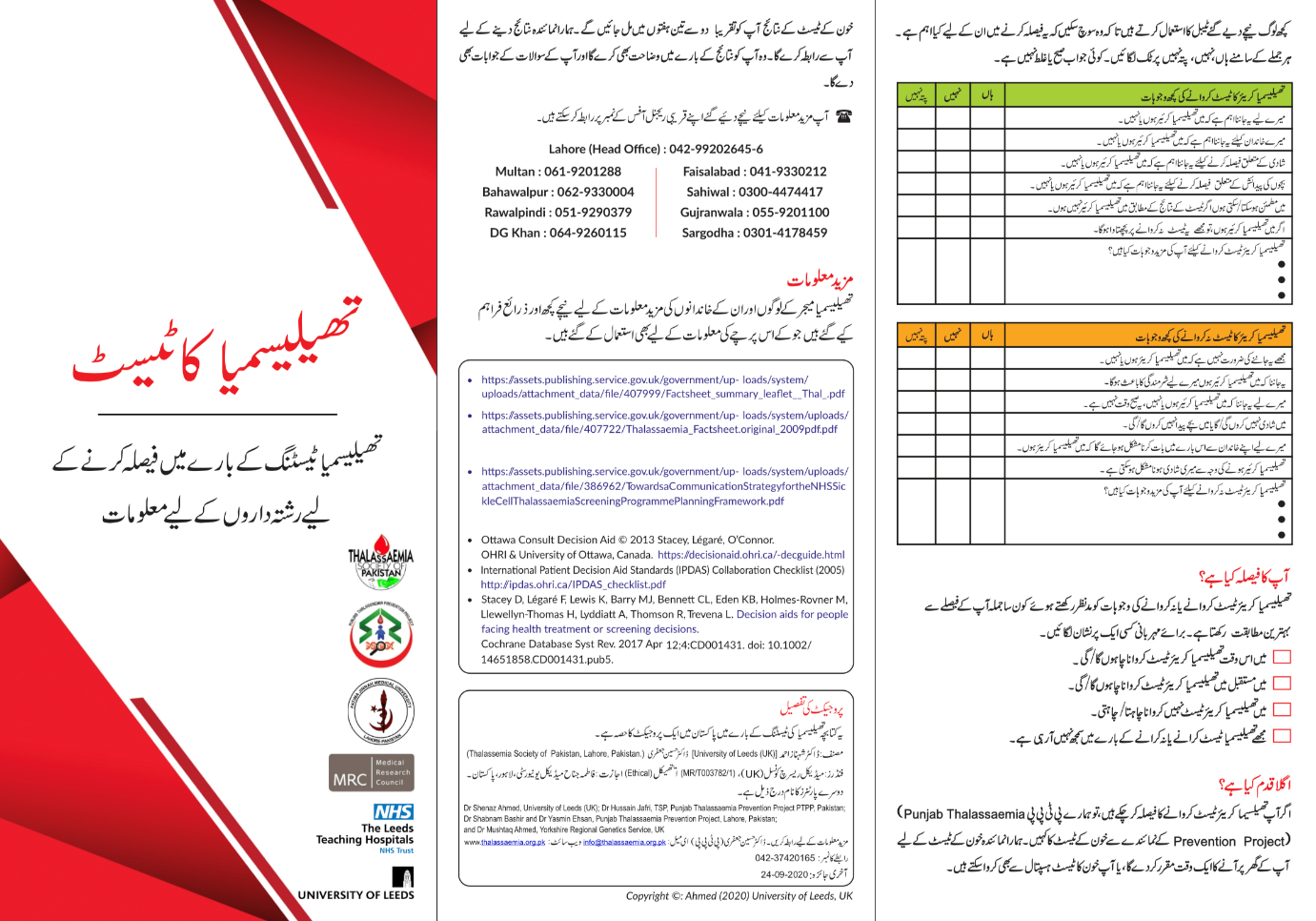

Supplement: Supplementary file 1 — The Decision Support Intervention for Relatives: Prototype [file 41431_2021_918_MOESM1_ESM.doc]
